# Purpuric Skin Rash in a Patient Undergoing Pfizer-BioNTech COVID-19 Vaccination: Histological Evaluation and Perspectives

**DOI:** 10.3390/vaccines9070760

**Published:** 2021-07-08

**Authors:** Gerardo Cazzato, Paolo Romita, Caterina Foti, Antonietta Cimmino, Anna Colagrande, Francesca Arezzo, Sara Sablone, Angela Barile, Teresa Lettini, Leonardo Resta, Giuseppe Ingravallo

**Affiliations:** 1 Section of Pathology, Departmente of Emergency and Organ Transplantation (DETO), University of Bari “Aldo Moro”, 70124 Bari, Italy; caterina.foti@uniba.it (C.F.); micasucci@inwind.it (A.C.); anna.colagrande@gmail.com (A.C.); angela.barile66@libero.it (A.B.); lettinit@yahoo.com (T.L.); leonardo.resta@uniba.it (L.R.); giuseppe.ingravallo@uniba.it (G.I.); 2 Department of Biomedical Science and Human Oncology, Dermatological Clinic, University of Bari, 70124 Bari, Italy; paolo.romita@uniba.it; 3 Department of Biomedical Sciences and Human Oncology, Gynecologic and Obstetrics Clinic, 70124 Bari, Italy; francesca.arezzo@uniba.it; 4 Section of Legal Medicine, Department of Interdisciplinary Medicine, Policlinico di Bari Hospital, University of Bari, 70124 Bari, Italy; sarasabloneml@gmail.com

**Keywords:** SARS-CoV-2, COVID-19, skin, rash, purpuric, vaccines

## Abstract

The COVID-19 pandemic has affected the entire planet, and within about a year and a half, has led to 174,502,686 confirmed cases of COVID-19 worldwide, with 3,770,361 deaths. Although it is now clear that SARS-CoV-2 can affect various different organs, including the lungs, brain, skin, vessels, placenta and others, less is yet known about adverse reactions from vaccines, although more and more reports are starting to emerge. Among the adverse events, we focused particularly on skin rashes. In this short report, we describe the case of a patient vaccinated with Comirnaty, who developed a purpuric rash resistant to oral steroid therapy after 2 weeks. To date, this is one of the very few cases in which skin biopsy was performed to better characterize the histopathological picture of this rash. Finally, we conduct a literature review of the cases of rashes from SARS-CoV-2 vaccines described in the literature, with the aim of laying foundations for future, larger case studies.

## 1. Introduction

The COVID-19 pandemic has affected the entire planet [1], and within about 1 and a half years, has led to 174,502,686 confirmed cases of COVID-19 worldwide, with 3,770,361 deaths [2]. In Italy, as of 6 December 2021, about 4.24 million confirmed cases, with 3.95 million recoveries and 127,000 deaths, have been recorded [3]. In the last few months, the advent of vaccines capable of effectively “educating” the immune system to respond to a possible COVID-19 infection has dramatically reduced infections, hospitalizations, and deaths, allowing many nations to start easing restrictions, with different time schedules and in various ways [4]. The new vaccines exploit the emerging “mRNA” technology rather than already established “vector” modes [5].

Although it is now clear that SARS-CoV-2 can affect various different organs, including the lungs [6], brain [7], skin [8,9,10], vessels [11], placenta [12,13] and others, less is known about adverse reactions from vaccines, although more and more reports are starting to emerge [14,15]. Among the adverse events, we focused particularly on skin rashes. In this short report, we describe the case of a patient vaccinated with Comirnaty, who developed a purpuric rash resistant to oral steroid therapy after 2 weeks. To date, this is one of the very few cases in which skin biopsy was performed to better characterize the histopathological picture of this rash. Finally, we conduct a literature review of the cases of rashes described in the literature after vaccines for other viruses and for SARS-CoV-2, with the aim of laying foundations for future larger case studies.

## 2. Materials and Methods

### Case Presentation

**Figure 1:**
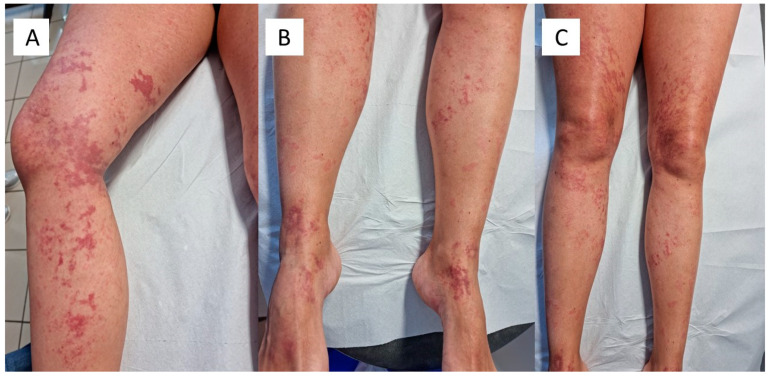
(**A**–**C**). Clinical spectrum of the purpuric rash in the lower limbs of the patient described. Note the different distribution of the lesions.

We present the case of a 43-year-old man who presented to the Dermatology and Venereology Complex Operative Unit for the appearance of an unspecified rash. At dermatological examination, the patient showed a purpuric rash on both lower limbs (Figure 1A–C), which had appeared about 15 days after the second administration of the Comirnaty mRNA vaccine. The patient had normal blood tests, apart from a slight rise in D-Dimer. A swab tested with Real Time-Polymerase Chain Reaction (RT-PCR) was negative, and a serological panel was also negative. His clinical history did not feature any allergies or diseases of note. With the patient’s consent, a 3.5 mm punch biopsy for in-depth diagnostics was decided upon, to allow the most appropriate therapy to be instituted. The biopsy specimens obtained were fixed in formaldehyde, buffered at 20% and sent to the U.O.C. of Pathology. After appropriate sampling, processing, inclusion in paraffin, and microtome cutting, histological sections about 5 microns thick were obtained for hematoxylin and eosin (H&E) staining. For immunohistochemistry purposes (IHC-P), other sections were incubated with SARS-CoV-2 spike protein S1 antibody (MA5-36247), rabbit monoclonal, isotype: IgG, at a concentration of 0.2 µg/mL, revealed with heat-mediated antigen in citrate buffer at pH 6. RT-PCR was performed.

The subsequent review of the literature was conducted on cases of patients who presented skin manifestations after different vaccination schedules. We referred to the electronic databases PubMed and Web of Science from the beginning of the administration of vaccines until 6 December 2021, using the terms “COVID-19 vaccines” or “nCov-19 vaccines” in combination with “skin” or “cutaneous manifestations”, or “eruption”, “rash” and “biopsy”, or “histopathology”, or “dermatopathology”, to see if any other cases of skin manifestations subjected to biopsy for histological analysis had been reported in the literature.

## 3. Results

**Figure 2:**
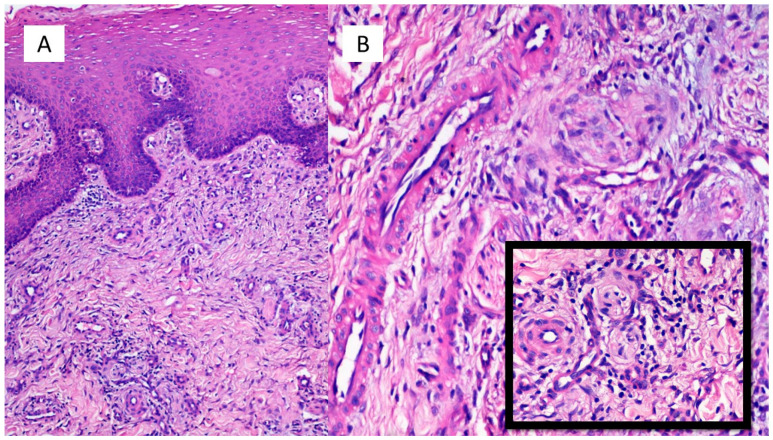
(**A**) Epidermis with modest hyper-parakeratosis, sometimes with a fusion of the contiguous epidermal ridges (Hematoxylin-Eosin, Original Magnification: 40×). (**B**) Evidence in superficial and middle dermis of blood vessels with endothelium often of the “hobnail” type, surrounded by a mild chronic inflammatory infiltration mainly constituted by lymphocytes and monocytes (Hematoxylin-Eosin, Original Magnification: 100×). Box: Detail of the phenomena of purpuric suffusion (Hematoxylin-Eosin, Original Magnification: 200×).

The histopathological examination showed an epidermis with modest hyper-parakeratosis, sometimes with fusion of the contiguous epidermal ridges (Figure 2A); involvement of the basal layer was not evident. At the level of the superficial and middle dermis, blood vessels with an endothelium of “hobnail” type were often seen, surrounded by a mild chronic inflammatory infiltrate mainly constituted by lymphocytes and monocytes (Figure 2B). Focal and alternating red blood cell extravasation in the dermis (purple) was rarely found (Figure 2 Box). Immunohistochemical reactions to SARS-CoV-2 spike protein S1 and PCR of biopsy tissue were both negative. The decision was made to start the patient on therapy with intravenous sodium methylprednisolone hemisuccinate at a dosage of 40 mg twice a day, for 7 days, gradually tapered until complete remission of the clinical picture.

## 4. Discussion

Since the advent of vaccines to prevent SARS-CoV-2 infection, there has been a reduction in infections worldwide [16]. As of 10 June 2021, a total of 2,156,550,767 vaccine doses have been administered, with 480 million completed vaccination schedules, achieving coverage of 6.2% of the world population [2]. In Italy, with 13.9 million completed vaccinations, 23.0% of the Italian population is covered [17]. Owing to the increased number of vaccines approved by regulatory bodies and hence increased number of doses administered, we are also witnessing a greater number of adverse events, that vary in type according to the different vaccination brands [18]. Krzysztof Rutkowsk et al. reported data that clearly demonstrate that anti-SARS-CoV-2 vaccines can both reduce the severity of the infection and prevent death. However, in their work, they showed that a small number of patients can experience anaphylaxis reactions. They have examined potential allergenic compounds in COVID-19 vaccines and describe an innovative allergy support model for vaccination centers which allows most patients with severe allergies to be immunized [19,20]. Among the various adverse reactions, some relating to the dermatological field have also recently been described. Devon E McMahon et al. report their experience from December 2020 to February 2021, recording a total of 414 skin reactions to the Moderna (83%) and Pfizer (17%) COVID-19 mRNA vaccines. Diffuse delayed local reactions were the most common, followed by local injection site reactions, urticarial eruptions and morbilliform eruptions. Among patients with reactions to the first dose, 43% experienced relapse at the second dose. Other less common reactions included pernio/chilblains, cosmetic filler reactions, shingles, herpes simplex flare-ups, and pityriasis rosea-like reactions [21]. The authors concluded that most patients with reactions to the first dose did not suffer a reaction to the second dose, and no serious adverse events developed in any of the patients in the registry after the first or the second dose. Their data support the concept that skin reactions to COVID-19 vaccination generally prove to be minor and self-limiting and should not discourage vaccination. In a letter to the editor, [22] Corbeddu M. et al. report their experience with 3170 healthcare workers vaccinated with the Pfizer-BioNTech COVID-19 vaccine, of which 0.91% (29 cases) developed mild adverse effects. Among these 29 cases, 38% (11 patients) developed skin symptoms, such as an erythematous-edematous reaction at the injection site, diffuse morbilliform rashes, mild erythema and positive dermographism. One patient, in addition to a mild urticarial rash, suffered exacerbation of his atopic dermatitis, previously well controlled by treatment with dupilumab. Four patients (36.3%) suffered extracutaneous manifestations such as laryngospasm, periorbital edema, and angioedema. All cutaneous manifestations resolved spontaneously within 2–3 days without treatment [22,23]. In a letter to the editor at the end of March 2021, Ring J. et al. summarized the adverse events recorded up to that time in cohorts of patients administered one of the available mRNA vaccines. More specifically, the authors [24] commented on the statement by the European Atopic Dermatitis Task Force (ETFAD), in which the putative risks of severe allergic reactions to COVID-19 vaccines for patients suffering from allergic skin diseases are discussed. Although systemic allergic reactions to vaccines are rare and mostly due to hypersensitivity to components of the vaccine formulation such as conjugating agents, preservatives, metals, stabilizers, adjuvants and contaminants [25], in the case of COVID-19 vaccines, in addition to mRNA, protein or vector, anaphylaxis could possibly be elicited by other ingredients, such as polyethylene glycol (PEG), present in the BioNTech/Pfizer (Comirnaty) and Moderna (mRNA-1273) vaccines. The authors conclude that based on the available data, the safety and tolerability of COVID-19 vaccines appear to be better than those of smallpox vaccines, for example [26,27]. Finally, they claim that nearly all patients with allergic skin diseases can be vaccinated with the registered COVID-19 vaccines available today. Precautionary measures should be taken in a very small subset of patients with a risk of a possible severe allergy to the vaccine ingredients. The knowledge of anaphylactic side effects is expected to grow among physicians and health care personnel in COVID-19 vaccination centers. More recently, in May 2021, Gronbeck et al. reviewed the current literature, observing that localized skin reactions were common following mRNA vaccines; among these, urticarial and morbilliform eruptions were the most frequent, but were rarely associated with anaphylaxis. There have been infrequent reports of herpes zoster, dermatological filler reactions, and immune thrombocytopenia, which mainly occurred in high-risk patient groups. The authors ultimately concluded that the skin reactions identified were largely self-limiting and should not discourage vaccination [28].

From the historical and histopathological point of view, histopathological changes in subjects subjected to vaccination against smallpox have been more amply characterized. In particular, the development of a papule that developed at the injection site, about 5 days later, is classically described [29]. The papule rapidly becomes vesicular and gradually dries, producing a crust that falls away, leaving a scar. Thanks to smallpox eradication, this vaccination is no longer administered. As a result, general vaccinia infection (so-called eczema vaccinatum), a serious complication of vaccination, is now of historical interest only [30]. In a 1993 paper, JR Miliauskas et al. described four cases of patients who had developed a single subcutaneous nodule at the site of a previous vaccine injection; three after the injection of the diphtheria, tetanus and pertussis vaccination and one after the tetanus toxoid vaccination. Presentation occurred with a mass at the injection site after 4–22 months from the vaccination. Histologically, three patients had a necrotizing granulomatous reaction with a surrounding infiltrate of lymphocytes, plasma cells, histiocytes and associated fibrosis. The fourth patient demonstrated a lymphohistiocytic reaction with a predominance of histiocytic cells and associated plasma cells, fibroblasts and fibrosis. Lymphoid infiltration in these reactions showed a predominance of T lymphocytes over B lymphocytes. Careful evaluation by the authors demonstrated the presence of aluminum in necrotic foci, inflammatory stroma, and granular cytoplasm of histiocytes. It was concluded that the reactions could have been immunological (hypersensitivity) reactions associated with the aluminum content in the preparation [31].

Furthermore, in recent months, an increasing number of reports relating to skin rashes, concomitant or a few days after the administration of anti-SARS-CoV-2 vaccines, has been reported. For example, Blumenthal et al. [23] reported the appearance of erythematous plaques, with a diameter measuring between 5 and 19 cm, and associated pruritus near or at the injection site. Skin biopsy revealed a superficial, perivascular and perifollicular lymphocytic infiltrates with rare eosinophils. The patients in question complained of fatigue, headache, chills. Reactions appeared approximately 8 days after the first dose and resolved on average after 6 days. The authors hypothesized a delayed-type or T-cell mediated hypersensitivity reaction [23]. Fernandez-Nieto et al. [32] and Johnston et al. [33] also reported pruritic and variably painful erythematous reactions near the injection site. The histological picture presented a mild, predominantly perivascular and focal interstitial mixed infiltrate with lymphocytes and eosinophils, consistent with a dermal hypersensitivity reaction. Additionally, in these cases, the possibility of a local delayed-type reaction to a component of the mRNA vaccine was evoked. Finally, Ackerman et al. reported an erythematous, pruritic injection site eruption which spread to the face, trunk, and extremities in patients vaccinated with mRNA vaccines. Skin biopsies showed a slight lymphocytic perivascular infiltrate [34]. Finally, in a letter to the editor in June 2021, Risa O. et al. showed that mRNA vaccine reactions tended to recapitulate the skin manifestations of SARS-CoV-2 positive subjects, making these data even more interesting having performed skin biopsies, with data similar to those presented in our paper [35].

To our knowledge, this is the first report of a skin biopsy performed in a subject who developed a purpuric-type rash two weeks after the second dose of Pfizer-BioNTech mRNA vaccine (as per delayed-type immunological reaction). The absence of allergies and/or pre-existing morbid conditions, concomitance with the vaccination administration and the negative results of immunohistochemical investigations for the S1 spike protein of SARS-CoV-2 and of RT-PCR on biopsy tissue suggest the likely association with the vaccine.

## 5. Conclusions

Our work relates to only one case, but it will be important in the future to report any new cases with these manifestations to try to broaden the polymorphous spectrum of skin manifestations and others likely evoked by SARS-CoV-2 vaccines. Nevertheless, in view of the normally self-limiting nature of such reactions, the most important point seems to be to ensure world coverage by vaccination against infection by this new pandemic agent.
